# Influence of Casting Solvents on CO_2_/CH_4_ Separation Using Polysulfone Membranes

**DOI:** 10.3390/membranes11040286

**Published:** 2021-04-13

**Authors:** Roba M. Almuhtaseb, Ahmed Awadallah-F, Shaheen A. Al-Muhtaseb, Majeda Khraisheh

**Affiliations:** Department of Chemical Engineering, Qatar University, Doha P.O. Box 2713, Qatar; roba.almuhtaseb@qu.edu.qa (R.M.A.); ahmed.awadallah@qu.edu.qa (A.A.-F.); s.almuhtaseb@qu.edu.qa (S.A.A.-M.)

**Keywords:** polysulfone (PSF), tetrahydrofuran (THF), chloroform (CF), membrane, gas separation

## Abstract

Polysulfone membranes exhibit resistance to high temperature with low manufacturing cost and high efficiency in the separation process. The composition of gases is an important step that estimates the efficiency of separation in membranes. As membrane types are currently becoming in demand for CO_2_/CH_4_ segregation, polysulfone will be an advantageous alternative to have in further studies. Therefore, research is undertaken in this study to evaluate two solvents: chloroform (CF) and tetrahydrofuran (THF). These solvents are tested for casting polymeric membranes from polysulfone (PSF) to separate every single component from a binary gas mixture of CO_2_/CH_4_. In addition, the effect of gas pressure was conducted from 1 to 10 bar on the behavior of the permeability and selectivity. The results refer to the fact that the maximum permeability of CO_2_ and CH_4_ for THF is 62.32 and 2.06 barrer at 1 and 2 bars, respectively. Further, the maximum permeability of CF is 57.59 and 2.12 barrer at 1 and 2 bars, respectively. The outcome selectivity values are 48 and 36 for THF and CF at 1 bar, accordingly. Furthermore, the study declares that with the increase in pressure, the permeability and selectivity values drop for CF and THF. The performance for polysulfone (PSF) membrane that is manufactured with THF is superior to that of CF relative to the Robeson upper bound. Therefore, through the results, it can be deduced that the solvent during in-situ synthesis has a significant influence on the gas separation of a binary mixture of CO_2_/CH_4_.

## 1. Introduction

Carbon dioxide (CO_2_) gas is the most common undesired impurity in natural gas (i.e., the major product is methane (CH_4_) gas), as its presence disturbs the content and values of energy and heat, triggering severe consequences such as pipeline corrosion and massive increases in the expenditures for transportation of gas. CO_2_/CH_4_ split-up is also imperative to be utilized as biogas, which is a replacement for natural gas [1,2,3]. Hence, the reduction of CO_2_ is considered a substantial development in the industries and has become the primary focus of numerous studies [1,2]. There are various methods for CO_2_ separation, such as absorption, cryogenic distillation, adsorption, and membrane separations [2]. Currently, the membrane separation technique for CO_2_/CH_4_ separation has been extensively studied in the field [4,5]. Undoubtedly, the membrane technology has a vital role in creating better optimization and production activities in industries, leading us towards sustainable and greener environment [6,7,8]. Microporous membranes and nonporous membranes are types of membranes utilized in separating gas mixture [9]. These types are divided into two subgroups, polymeric membranes, which are usually nonporous membranes and recognized as rubber membranes with high permeability with low selectivity, and glassy membranes with superior selectivity but low permeability [10].

Further, the polymeric membranes especially have obtained the attention of the international scientific community for gas separation due to unique features such as scale-up ability, flexibility, low cost, low energy consumption, ease of operation, and eco-friendliness [11,12]. Additionally, there are two key variables of membranes that affect their performance. 

The sublayer formation of the asymmetric membrane was controlled by several paramters in the casting solution such as composition, coagulation temperature, and organic and inorganic additives [13]. Further, the formation of asymmetric membranes was also affected by the polymers type, solvents, and nonsolvents utlized [14].

Moreover, it is well known that the properties of solvent have an effect on the morphology and performance of the membrane operation. For example, addition of a volatile solvent into a polymeric solution, along with a non-solvent, can change liquid liquid demixing behavior, and as a result can cause a change in the membrane morphology and performance [15]. Furthermore, adding a co-solvent to a polymeric solution can eliminate macro-void formation during instantaneous demixing and change the morphology of the membranes from finger-like to sponge-like structure, despite instantaneous demixing [16]. The main advantages of membrane technology are related coherently to the transport selectivity and permeability of the membrane used [17].

These parameters are permeability and selectivity [18]; however, the optimization procedure is an immense challenge to develop the membranes and to use them for separating the gas mixture. Further, it is clearly understood that high permeability denotes a low selectivity, and vice versa [19]. The relationship between permeability and selectivity is conducted exploiting plots of Robeson upper bound generated by Robeson (1991) that are defined as a target for optimizing the permeability and selectivity [19,20,21,22]. The composition of the gas mixture and permeability are significant specifications that determine the separation of gas mixtures for the plots [23]. Robeson upper limit correlation is depicted in the below formula:

Pi = kα^n^_ij_ (1)

where Pi is the permeability for the rapid gas, k indicates to the front factor, αij indicates to the separation factor, and n is the slope (m) of the plot [21]. Moreover, αij is the selectivity (separation factor) for i/j gas mixture [24]:

αij = (P_i_)/(P_j_) (2)

Huge groups of polymers were used to prepare membranes, including matrimid ^®^, polysulfones (PSF), polyethersulfone (PES), and rubbery polymers like polyethylene glycol, PEBAX^®^, PDMS, and polyurethanes (PUs), etc. [18,25]. It is noteworthy to mention in this context that the PSF are widely used for membrane fabrication due to their low costs and high thermal and chemical resistance to solvents. Moreover, they have comparatively a long lifetime, high hydrophobicity, excellent mechanical radical oxidation stability, as well as high resistance to swelling in concentrated acids. It is noted that they also form porous asymmetric structures [26,27]. Additionally, various solvents have been used to cast membranes for gas separation processes such as chloroform, tetrahydrofuran, dimethylacetamide, and N-methyl-2-pyrrolidone, methanol, ethanol, dimethylformamide, isopropyl alcohol, hexane, dimethylsulfoxide, benzene, etc. [19]. This selection of solvents should require several pivotal requirements, specifically their volatility and ability to dissolve the respective polymer [19,25]. It is reported that the ideal selectivity for CO_2_/CH_4_ was ~25.5, and permeability values for single gases of CO_2_ and CH_4_ were 0.51 and 0.02 barrer, correspondingly at a pressure of 3.5 bar and temperature of 25 °C when PES is used with NMP as a solvent [11]. Alternatively, another investigation observed the impact of the feed temperature and pressure on the selectivity and permeability of a PSF membrane synthesized by dimethylacetamide as solvent. Subsequently, the results displayed that the permeabilities of CO_2_ and CH_4_ at 5 bar were 7.13 and 0.24 gas permeation unit (GPU), respectively, while the selectivity was stated as 29.7 [20]. Another investigation prepare a PSF-based membrane employing chloroform. The permeability of CO_2_ and CH_4_ at 10 bar and 22 °C was 6.9 and 0.28 barrer, accordingly, whereas SCO_2_/CH_4_ was 25 [27]. It was testified that when N-methyl-2-pyrrolidone was used as a solvent with Matrimid^®^, the results declared that the ideal CO_2_/CH_4_ selectivity was 28.6, the permeability of CO_2_ was 5.72 barrer, and the permeability of CH_4_ was 0.2 barrer at 4 bar and 35 °C [28]. Furthermore, CO_2_/CH_4_ selectivity value 12.3, was obtained using a polymer of intrinsic microporosity (PIM) membrane that was generated with chloroform in a CO_2_/CH_4_ separation at 2 bar and 30 °C [29]. It was reported that decreasing the temperature had a substantial influence on the permeability of CO_2_ when the same material and pressure were utilized; however, a lower temperature of 25 °C was required to carry out the procedure [30]. The main objective of this study is to fabricate PSF membranes via supporting two different casting solvents: chloroform and tetrahydrofuran. Various techniques will be used to characterize the synthesized membranes utilizing the different conditions. Further, the study will tackle the influence of the feed pressure and evaporated solvent-based-PSF structure on the two main characteristics: permeability and selectivity of CO_2_ and CH_4_ gases as well.

List of abbreviations used in the text are listed below in Table 1.

## 2. Experimental Methods

### 2.1. Materials

Three main chemicals; polysulfone (Mw = ~22,000; density = 1.24 g/mL; Sigma Aldrich, St. Louis, MO, USA), chloroform (99.8%; density = 1.49 g/mL; Sigma Aldrich, St. Louis, MO, USA), and tetrahydrofuran (99%; density = 0.889 g/mL Riedel-de Haën, Germany) were used as received. The CO_2_/CH_4_ gas mixture (5% CO_2_ and 95% CH_4_) and Helium (99.999%) were supplied from Buzuair Scientific and Technical Gases, Qatar.

### 2.2. Membrane Preparation

**Figure 1:**
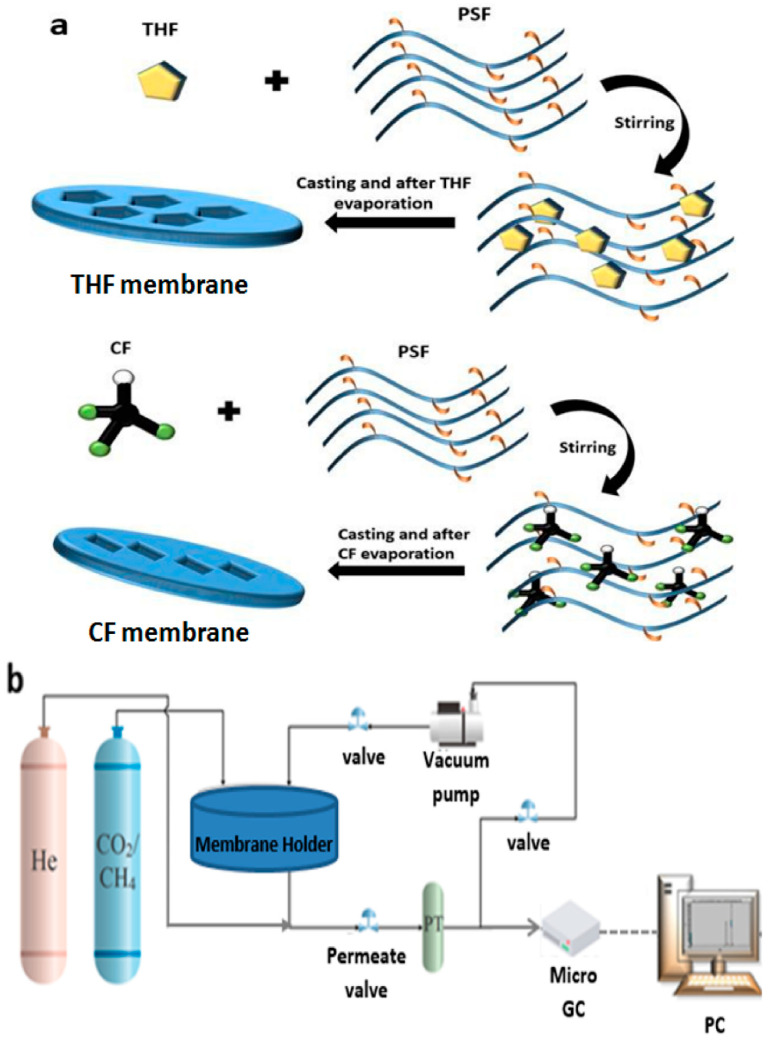
Schematics of (**a**) the shaping of PSF membranes by different solvents (i.e., THF and CF), (**b**) schematic system setup of CO2/CH4 gas mixture separation.

Two different casting solvents of CF and THF were selected because of their capability to dissolve PSF. About 5 g quantity of PSF was added to 19 milliliters of each solvent in a separate glass container (100 milliliters) and stirred at ~30 °C for 24 h to obtain a homogeneous dissolved solution. The total concentration of PSF in each solvent is 0.3 g/mL. An Elcometer (3700 Doctor Blade, Belgium) was used to cast the resultant solution on glass plates (30 × 21 cm^2^) to prepare the sheets of the membrane. Further, CF- and THF-casted membranes were dried at ~20 °C for 24 h. The thickness of the outcome membrane was conducted using a thickness gauge (0.001 mm Electronic Thickness Gauge, 10 mm Digital Micrometer) that had a range from ~85–200 µm. Moreover, the suggested mechanism of shape of the structural forming for PSF before and after evaporation process of solvent utilized is depicted in Figure 1a.

### 2.3. Characterization of the Membranes

FTIR spectra were used on a Jasco spectrophotometer for identification of those prepared with range of 4000 to 400 cm^−1^. The FT-Raman spectra were collected by a Bruker FT-Raman spectrometer of type RFS 100/S attached to a Bruker-IFS 66/S spectrometer. The morphology of the membrane was observed with an FEI Nova^TM^ NanoScanning Electron Microscope 450 (Nova NanoSEM). Thermogravimetric analyses (PerkinElmer Pyris 6 TGA) were conducted under nitrogen gas flow with a ramp of 10 °C/min from ambient temperature to 850 °C. X-ray diffraction (XRD) measurements were also performed using a Miniflex II Benchtop XRD analyzer, manufactured by Rigaku Corporation Japan. The 2θ scan data were collected over the range of 5 to 90° with scan rate 5°/min.

### 2.4. CO_2_/CH_4_ Separation

A flat sheet membrane setup was utilized to testify the performance of the PSF membranes for separating CO_2_/CH_4_ gas mixture, as exposed in Figure 1b. Initially, the membrane was positioned into the system after cutting it into a proper size. The setup was remained overnight to make sure it was free from any contaminants from surrounding gas or solvent residues. The gas mixture was fed at a prearranged pressure ranging from 1 to 10 bar for 3 h at room temperature to obtain the equilibrium. Afterward, the permeate sample was collected for Micro-GC analysis (Agilent Technologies-490 Micro GC, Agilent Technologies, Inc. Headquarters, US). The pressure and temperature of permeate were proceeded using a pressure–temperature transducer with a precision of 0.05%. Since the permeate pressure was low compared to the Micro-GC requirements, the permeate side of the setup was charged with He (inert) gas, as a carrier gas with a pressure of ~1.8 bar. From the Micro-GC analysis, the compositions of the gases in permeate were measured and used for the selectivity and permeability calculations, as explained in the Supplementary Data File.

## 3. Results and Discussion

### 3.1. Characterization

**Figure 2:**
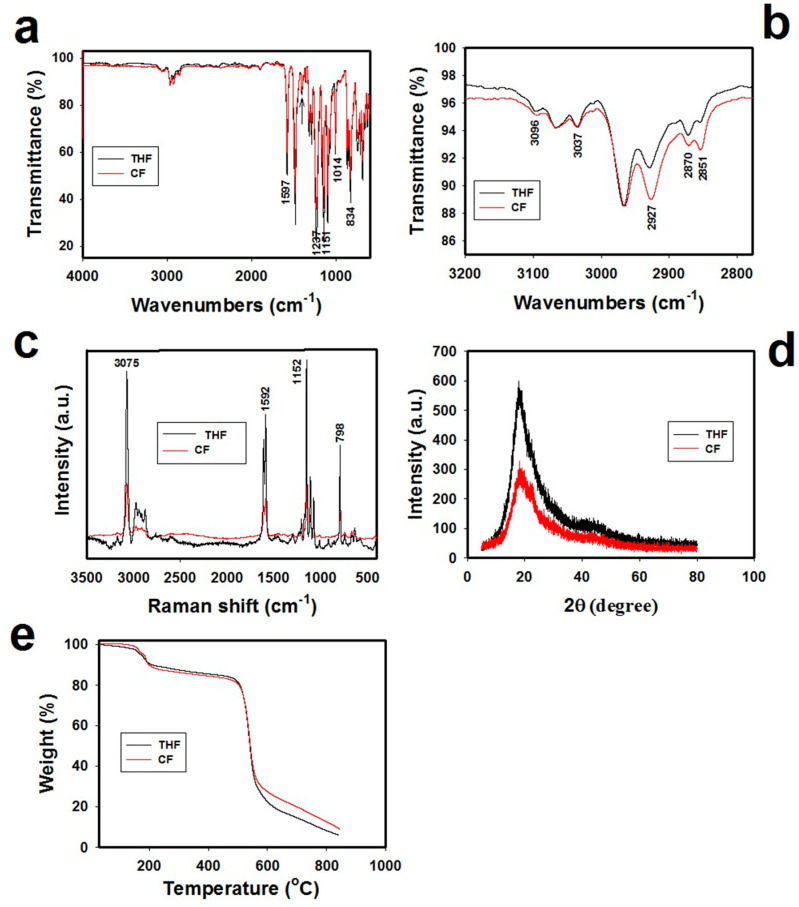
(**a**) Full scale of FT-IR spectra from 4000 to 500 cm^−1^, (**b**) zoomed-in scale from 3200 to 2800 cm^−1^, (**c**) Raman spectra, (**d**) X-ray diffraction patterns, and (**e**) TGA (on the left side of y-axis)&DTG (on the right side of y-axis)curve s of the PSF membranes prepared using THF and CF.

The Fourier Transform Infrared Spectroscopy (FT-IR) spectra images for the PSF membranes formulated using THF and CF as solvents are shown in Figure 2a. It is displayed that the fingerprint peaks for the PSF membrane appeared at 1151cm^−1^ (O-S-O stretching), 1237 cm^−1^ (C-O-C stretching), and 1597 cm^−1^ (C–C aromatic). To elaborate more, the peaks occurring at 1014 and 834 cm^−1^ refer to C–H stretching for the aromatic ring in PSF. 

Figure 2b shows the FT-IR region from 3200 to 2800 cm^−1^, specifically, as the variances and alterations in the significant peaks emerge for the membranes. Moreover, there are some peaks of THF and CF-casted membranes appeared at 2851, 2870, 2927, 3037, and 3096 cm^−1^. The peaks of 2851, 2870, and 2927 refer to C-H stretching in the alkene group in THF-casted membrane or CF-casted membrane as well [31]. Overall, it can be seen that intensities of THF peaks are higher than the intensities of CF peaks.

Raman spectra of the PSF membranes conducted using THF and CF solvents are illustrated in Figure 2c. The four peaks at ~798, 1152, 1592, and 3075 cm^−1^ relate, accordingly, to the asymmetric C-S-C, asymmetric C-O-C, aromatic ring chain, and C–H vibrations [29]. The intensities are at these four peaks for THF-casted membrane is higher than the intensities of these four peaks of CF-casted membrane. Therefore, the Figure 2c reveals that THF does not affect the Raman shift of PSF, unlike the CF that affects the PSF Raman shifts strongly, and these changes support the assumption in Figure 1a. The X-ray diffraction (XRD) patterns that are depicted in Figure 2d referring to that PSF membranes fabricated using THF and CF had almost similar patterns. These outcomes of the results are in agreement with reported works [32,33,34]. However, the membrane casted with THF exhibits a higher full width at half maximum than that of CF. This confirms that using different solvents has a well-defined effect on the internal physical structure for the PSF membranes, as proposed in Figure 1a.

Achieving stability in temperature is considered a great challenge in the membrane process for gas separations, especially when extreme temperatures are utilized [35]. The thermal gravimetric analysis (TGA) results are shown in Figure 2e. These results report that the PSF membranes casted with both solvents were thermally stable up to 580 °C, with a successive and rapid weight loss down to ~50%. Therefore, the thermal decomposition curves from the derivative weight (DTG) for the two membrane samples exposing the two major weight losses are around 200 and 580 °C. Consequently, the major weight losses are assignable to the degradation side function groups and of the backbone of PSF, respectively [36]. Likewise, the weight losses occurring at the start of the TGA plot represent that there is the existence of some type of moisture present in the membrane that can result in affecting the separation efficiency of the membrane [37,38,39]. Moreover, it is also seen that the membrane casted with CF gives slightly better thermal stability than the one casted with THF.

**Figure 3:**
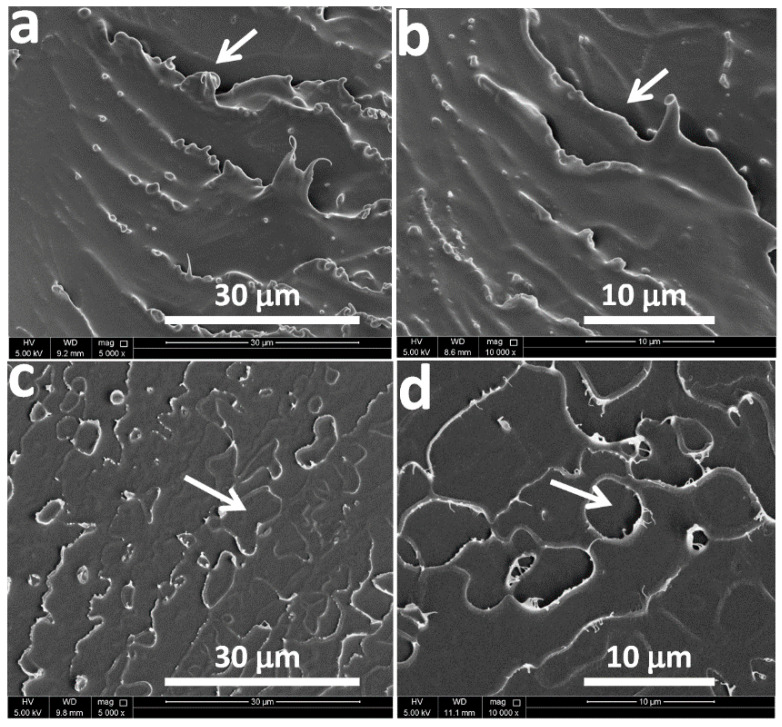
SEM photomicrographs of casted membranes by CF and THF at different amplifications. (**a**,**b**) refer to CF at bar scale 30 and 10 µm, and (**c**,**d**) refer to THF at bar scale 30 and 10 µm. The arrows refer to the morphology change.

Figure 3a–d is a representation of the SEM photomicrographs of casted membranes by two different solvents utilized in the study: CF and THF, accordingly. Generally, for pure PSF membrane, a porous uniform structure typically like sponge is noticed, as mentioned in innumerous reported studies [40,41,42]. Further, Figure 3 below refers to some difference in the morphology and structure of two membranes due to the evaporation of solvents during drying process. The arrows in Figure 3a,b signify the different amplification of CF membranes: 30 and 10 µm, respectively. Further, the structure seems like grooves and relatively deep pores on the surfaces, as pointed to by arrows. Additionally, Figure 3c,d refers to THF membrane at two different amplification: 30 and10 µm, respectively. The arrows in the below figures denote grooves that occur only at the surface, unlike CF membranes, where the grooves appear deeper. Moreover, the disparity of morphology between the CF and THF membranes is noticeable. Hence, this variation in morphology can have a significant effect on the gas separation process of CO_2_/CH_4_. Consequently, these observations concluded from SEM are in agreement with assumption of solvent-based morphology variation formation in Figure 1a. This is due to the difference in physical and chemical properties of each solvent.

### 3.2. CO_2_/CH_4_ Separation

**Figure 4:**
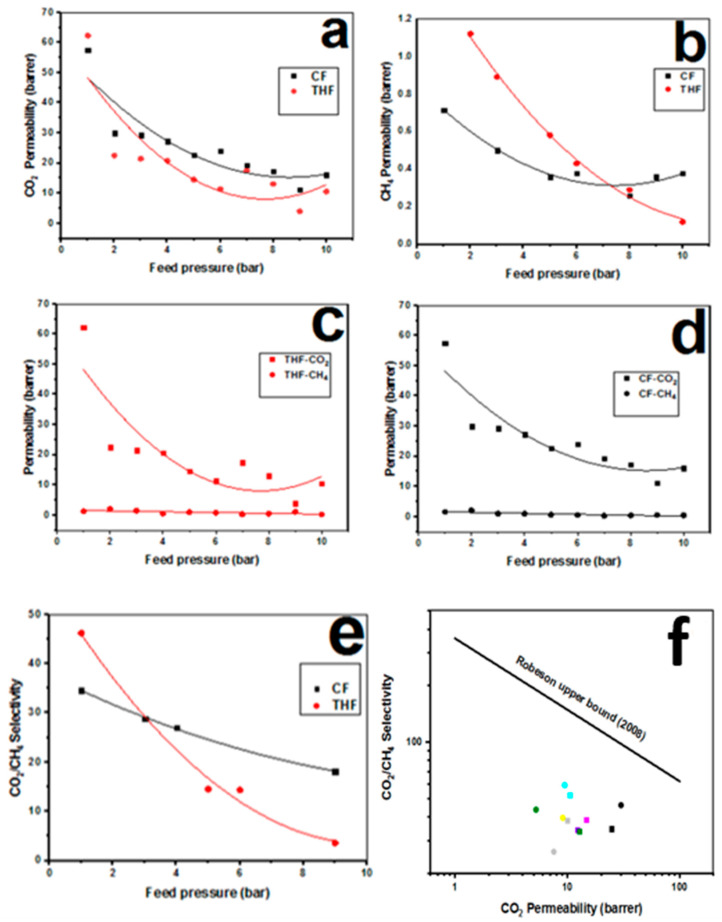
(**a**) CO2 permeabilitie s from THF and CF, (**b**) CH4 pe rme abilitie s from THF and CF, (**c**) permeabilities of CO2 and CH4 for THF, (**d**) permeabilitie s of CO2 and CH4 for CF, (**e**) CO2/CH4 selectivitie s for THF and CF, and (**f**) selectivity value s of CO 2/CH4 for THF (Symbols **•**, **•**, **•**, **•** and • refer to 1, 4, 7, 8, and 10 bar, respectively) and CF (Symbols **■**, **■**, **■**, **■**, **■** and **■** refer to 1, 5, 6, 7, 8, and 10 bar, respectively) in Robeson upper bound limit.

Figure 4a–f refers to the permeability and selectivity of CO_2_/CH_4_ (S_CO2/CH4_) through PSF membranes synthesized by THF and CF solvents. Figure 4a,b illustrates the permeability for CO_2_ and CH_4_ gases, respectively. It is seen from these two figures that the permeability values for CO_2_ and CH_4_ declined as the feed pressure is increased. 

This is due to flexibility of membrane pore structure. Nonetheless, membrane of THF displayed a more rapid decrease than that of CF membrane at the same corresponding pressure value. Further, with the results, it can be deduced that the highest values for CO_2_ and CH_4_ gases are obtained at low feeding pressures. The nature of the polymer used and the dual-mode sorption may be responsible for this behavior [20]. In conclusion, it could be said that the permeability of CH_4_ gas is low and approximately constant in comparison to permeability of CO_2_ gas for both membranes prepared using CF and THF as solvents, as exposed clearly in Figure 4c,d. Figure 4c,d interprets the permeability of CO_2_ and, CH_4_ gases for the casted membranes by solvents THF and CF, respectively. This deviation of permeability in both Figure 4c,d is attributed to the molecular weight, molecular shapes of CO_2_ and CH_4_ gases, and their dipole moments [12]. Figure 4e shows the relation between the selectivity for CO_2_/CH_4_ through PSF membranes that are prepared by THF and CF as solvents with respect to the feeding pressure ranged from 1 to 10 bar. Therefore, it can be deduced that the highest selectivity obtained at low feed pressure in both membranes casted with THF and CF. This behavior could be because when high pressure is applied on the membrane, it causes surface defection, and this affects the adsorption mechanism of the membrane. Consequently, this affects the penetration of CO_2_ and CH_4_ molecules without being selective. Another consequence of exposing the membrane to high pressure is that it can have an influence on the free volume in the membrane [42]. Thus, as shown in Figure 4e, the S_CO2/CH4_ values decrease when the pressure augments. However, this decline is more noticeable in THF. The S_CO2/CH4_ of CF is illustrated to be superior to the S_CO2/CH4_ value of THF when the feed pressure is >2 bar. Moreover, the S_CO2/CH4_ of THF-casted membrane approaches to zero by increasing pressure. Therefore, it can be deduced that the best selectivity is at 1 bar for both membrane solvents. Polymeric membranes for gas separation show a trade-off between gas permeability and selectivity as confirmed by Robeson with his upper bound curve in 1991 [43] and later modified in 2008 [21], as mentioned previously. Figure 4f depicts the performance of separation process of these membranes in a Robeson upper bound limit for CO_2_/CH_4_ [21]. It was perceived from the plot that the closest points to the Robeson limit were at 7 bar for both THF and CF membranes. Furthermore, it was noticed that THF is a little closer to the Robeson upper bound limit, which refers to an enhanced performance. Hence, it can be deduced that this behavior indicates to better performance of THF and CF membranes that might be due to the generated pores that arose upon THF molecules evaporation from membranes matrices during the drying process, as suggested in Figure 1a. 

Further, Table 2 below refers to a comparative review of the performances attained in this work with a deep comparison to those reported in the literature. Overall, it can be noticed that that the CO_2_/CH_4_ selectivity using PSF membrane and THF and CF as casting solvents is higher compared with other membranes. It can be observed that the present study reports the highest selectivity for CO_2_/CH_4_ for PSF with THF with a value of 50. Although the permeabilities of CO_2_ and CH_4_ presented in this study fluctuate between low and high with comparison to reported values, the regression value >0.9 indicates high efficiency of the displayed membranes.

**Table 2:** Comparison of CO_2_/CH_4_ mixed gas selectivity and permeability values with those from literature.

**Regression**							
**Solvent**		**CO_2_/CH_4_ Selectivity**		**CO_2_ Permeability**		**CH_4_ Permeability**	
THF		y = 0.51 x^2^ − 10.40 x + 55.87,		y = 4.96 x^2^ − 0.07 x + 0.31,		y = 9.14 x^2^ − 2.18 x + 0.013,	
		R^2^ = 0.98		R^2^ = 0.95		R^2^ = 0.99	
CF		y = 0.11 x^2^ − 3.13 x + 37.56,		y = 2.75 x^2^ − 0.03 x + 0.20,		y = 7.27 x^2^ − 1.08 x + 6.42,	
		R^2^ = 0.99		R^2^ = 0.91		R^2^ = 0.95	
**Membrane**	**Solvent**	**CO_2_/CH_4_ Selectivity**	**CO_2_ Permeability (barrer)**	**CH_4_ Permeability** **(barrer)**	**∆P** **(bar)**	**T** **(°C)**	**Ref.**
PSF	tetrahydrofuran	50	30.04	0.65	1	20	Present work
PSF	chloroform	35	24.76	0.715	1	20	Present work
Poly (vinylidene fluoride) (PVDF)	N-methyl-2-pyrrolidone	26.37	2.11	0.08	7	35	[1]
Matrimid/PVDF (3%)	N-methyl-2-pyrrolidone	42.81	9.42	0.22	7	35	[1]
Matrimid	chloroform	31	20	0.5	3	25	[2]
PES	N-metthyl-2-pyrrolidone	25.5	0.51	0.02	3.5	25	[11]
PES	dimethylacetamide	29.7	7.13	0.24	5	30–70	[20]
PSF	chloroform	25	6.9	0.28	10	22	[27]
6FDA-bisP	chloroform	27	30	2	5	25	[44]
Matrimid	dichloromethane	31.13	7.16	0.23	4	35	[45]
Matrimid	N-methyl-2-pyrrolidone	28.6	5.72	0.2	4	35	[28]

## 4. Conclusions

This study presents the effect of two different casting solvents CF and THF to synthesize PSF membranes. The result showed that these membranes have a significant influence on the separating process of CO_2_/CH_4_ mixture. Further, the results also revealed that using THF as casting solvent exposed better performance in the separation process of CO_2_/CH_4_ gases compared to CF membranes by applying Robeson upper bound technique. The results indicated to that by augmenting the feed pressure value, this affected adversely on both factors, permeability and selectivity, for both membranes obtained with different solvents. Furthermore, it was deduced that when THF was utilized to prepare the membrane, the values of permeability for CO_2_ and CH_4_ gases were approximately 62.32 and 2.06 barrer at 1 and 2 bars, respectively. While, for CF, the values of permeability for CO_2_ and CH_4_ were 57.59 and 2.12 barrer at 1 and 2 bars, respectively. Moreover, the selectivity values were maximum at 1 bar for both membranes at 48 and 36 for THF and CF, respectively. This concludes that the performance of the membrane casted with THF is better compared to the membrane casted by CF. Therefore, it can be said that both of CF and THF solvents during casting of PSF membrane have a significant impact to separate CO_2_/CH_4_ gases of binary mixtures and have the potential to be used in large scale.

## Figures and Tables

**Table 1:** List of abbreviations.

Full Name	Abbreviation
Chloroform	CF
Tetrahydrofuran	THF
Polysulfone	PSF
Gas Permeation Unit	GPU
Polyethersulfone	PES
Polydimethylesiloxane	PDMS
Thermogravimetric analysis	TGA
X-Ray Diffraction	XRD
Gas Chromatography	GC

## Data Availability

Not applicable.

## Supplementary Materials

The following are available online at https://www.mdpi.com/article/10.3390/membranes11040286/s1.
