# Supplementary material for: Influence of Casting Solvents on CO2/CH4 Separation Using Polysulfone Membranes
Source: Membranes (Basel). 2021 Apr 13;11(4):286. doi: 10.3390/membranes11040286 (PMC8070651; doi:10.3390/membranes11040286)
Supplement: Supplementary file 1 [file membranes-11-00286-s001.pdf]

membranes

MDPI

Supplementary Data File

Influence of Casting Solvents on CO_2/CH_4 Separation using Polysulfone Membranes

Roba M. Almuhtaseb, Ahmed Awadallah-F †, Shaheen A. Al-Muhtaseb and Majeda Khraisheh *

Department of Chemical Engine e ring, Qatar University, Doha, P.O. Box 2713, Qatar; roba.almuhtaseb@qu.edu.qa, ahmed.awadallah@qu.edu.qa, s.almuhtaseb@qu.edu.qa

* Correspondence: m.khraisheh@qu.edu.qa; Tel.: +974-4403-4993; Fax: +974-4403-4131

† On leave from the Radiation Research of Polymer Department, National Centre for Radiation Research and Technology, Atomic Energy Authority, P.O. Box 29, Nasr City, Cairo, Egypt.

* Correspondence: e-mail@e-mail.com; Tel.: (optional; include country code; if there are multiple corresponding authors, add author initials)

S1. Theory

The Equations (1) and (2) are used to calculate the permeability of the membrane as follows:

$$J_i = \frac{n_i}{t.A} \quad (1)$$

$$P_i = \frac{J_i.l}{\Delta P_i} \quad (2)$$

where J_i is the flux, $\frac{cm^3}{s.cm^2}$, n_i is the molar volume at standard temperature and pressure (cm^3), t is the experiment duration (s), A is the cross surface area of membrane (cm^2), P_i is the permeability, $\frac{cm^3.cm}{s.cm^2.cmHg}$, but the unit of permeability is known as $barrer$, l is the thickness of membrane (cm), and ΔP_i is the partial pressure difference of each gas,($cm\ Hg$). Equation (3) is used to calculate the selectivity of CO_2 with respect to CH_4 as follows:

$$\alpha = \frac{P_{CO_2}}{P_i} \quad (3)$$

Where i represents CH_4. The data in **Table S1** are needed to calculate the permeability and selectivity values.

Table 1. Data needed for calculations.

Parameters.	
**Mole fraction of CH_4 in feed, $yCH_{4,feed}$=**	0.95
Mole fraction of CO_2 in feed, $yCO_{2,feed}$=	0.05
Thickness of membrane casted by CF, $\boldsymbol{l}$ =	0.0132 cm
Thickness of membrane casted by THF, $\boldsymbol{l}$ =	0.0116 cm
Permeate volume, V =	92 cm^3
Cross surface area of membrane, A =	14.52 cm^2
Duration of experiment, t =	10800 s
Atmospheric pressure, P_{atm} =	1.013 bar
Gas constant, R =	83.1447 $\frac{bar.cm^3}{mole.K}$
Operating temperature, T=	293.15 K
Pressure of Feed, P_{feed}=	1-10 bar (gage)
Pressure of Helium, P_{He}=	1.5-2 bar

Pressure of Permeate, P_{perm}=	The pressure of the sample taken from GC bar
Total pressure (P_{He}+ P_{perm}), P_{Totat} =	bar
Density of membrane =	$\sim 1.24 g/cm^3$

Calculating number of moles of Helium as follows:

$$n_{He} = \frac{P_{He}V}{RT} \quad (4)$$

Calculating the total number of moles as follows:

$$n_{Total} = \frac{P_{Total}V}{RT} \quad (5)$$

Calculating number of moles in permeate as follows:

$$n_{Perm} = \frac{P_{Perm}V}{RT} \quad (6)$$

Calculating number of moles of CO_2 in permeate as follows:

$$n_{CO2} = n_{Perm} \times y_{CO2,feed} \quad (7)$$

Calculating the mole fraction of CO_2 in permeate as follows:

$$y_{CO2,perm} = \frac{n_{CO2}}{n_{Perm}} \quad (8)$$

Calculating the partial pressure difference of CO_2 as follows:

$$\Delta P_{CO2} = y_{CO2,feed} \times P_{feed,Abs} - y_{CO2,perm} \times P_{Total} \quad (9)$$

Calculating the flux of CO_2 using Equation (10) as follows:

$$J_{CO2} = \frac{n_{CO2}}{t.A} \quad (10)$$

Same procedures will be done to find the permeability of CH_4

The selectivity of CO_2 is calculated using Equation (11) as follows:

$$\alpha = \frac{P_{CO_2}}{P_{CH4}} \quad (11)$$
